# Toxicological Assessment of Toxic Element Residues in Swine Kidney and Its Role in Public Health Risk Assessment

**DOI:** 10.3390/ijerph6123127

**Published:** 2009-12-08

**Authors:** Dragan R. Milićević, Milijan Jovanović, Verica B. Jurić, Zoran I. Petrović, Srđan M. Stefanović

**Affiliations:** 1 Institute of Meat Hygiene and Technology, Kaćanskog 13, 11000 Belgrade, Serbia; E-Mails: zoran@inmesbgd.com (Z.I.P.); ssrdjan@inmesbgd.com (S.M.S.); 2 Department of Pathomorphology, Faculty of Veterinary Medicine, University of Belgrade, Bulevar Oslobođenja 18, 11000 Belgrade, Serbia; E-Mail: milijan@vet.bg.ac.rs (M.J.); 3 Department for Animal Sciences, Faculty of Agriculture, University of Novi Sad, Trg Dositeja Obradovića 10, 21000 Novi Sad, Serbia; E-Mail: vjuric@polj.fac.ac.rs (V.B.J.)

**Keywords:** toxic elements, kidney, residue, pathomorphology, swine

## Abstract

In order to ensure the safety of consumers in Serbia the prevalence of toxic elements (As, Cd, Hg, Pb) in swine kidney collected from three different areas in Serbia (n = 90) was determined by atomic absorption spectrometry. Also, in order to find information on the effects of accumulation of toxic elements on swine kidney, pathohistological examination of the kidneys was performed. The presence of mercury was found in 33.3% of kidney samples in the range of 0.005–0.055 mg/kg, while the presence of cadmium was detected less often (27.7%) but in larger amounts (0.05–1.23 mg/kg). The presence of arsenic was found only in one sample, while no lead was found. The results of the metal-to-metal correlation analysis supported there were the result of different sources of contamination. Pathohistological examination of kidneys confirms tubulopathies with oedema and cell vacuolization. In addition, haemorrhages and necrosis of proximal kidney tubule cells were found. This study demonstrates that toxic elements in Serbian slaughtered pigs are found at levels comparable to those reported in other countries, and consequently the levels reported in this study do not represent a concern from a consumer safety point of view. The lack of a strong correlation between histopathological changes and the incidence of toxic elements found in this study might be explained as the result of synergism among toxic elements and other nephrotoxic compounds which enhance the toxicity of the individual toxins even at the relatively low mean concentrations observed in this study.

## 1. Introduction

Environmental pollution with toxic elements is a dangerous problem that is recognized worldwide. Toxic elements can be found in water, soil, air, plant and animal tissues as a result of both natural causes, industrial and agricultural practices [1]. Environmental concern pertaining to toxic elements relates to their toxicity, labile nature, bioaccumulation in organisms and ultimately to their effect on human beings [2]. One of the most important aspects of environmental pollution for humans is the intake of toxic elements in the diet [3]. Heavy metals are significantly toxic, due to their cumulative nature in the different body organs leading to unwanted effects [4,5]. Metals tend to bioaccumulate in the environment and biomagnify in food chains [6], their levels might reach toxic limits even when found in low concentrations in environmental samples. Since this should be limited to an unavoidable minimum, much attention is paid to the occurrence of these elements in food. Monitoring programmes are being carried out in many countries with the purpose of avoiding the distribution of foodstufs that could pose a risk to human health if consumed. The chemical interactions of toxic elements with proteins in living systems render essential reactions impotent [7]. Toxic elements can interfere with the functions of enzymes and are responsible for many diseases, especially cardiovascular, renal, nervous and even bone disorders [4,8–12]. Toxic elements are also implicated in several major human diseases including carcinogenesis-induced tumor promotion [13–17]. Some toxic elements are considered to be carcinogenic, mutagenic and teratogenic in experimental animals [18–19].

Toxic elements levels in animal tissues are organ specific. Meat and meat products form an important part of the human diet. Although the toxic elements content in muscle is generally low, offals, such as liver and kidney, often contain higher metal concentrations than most other foods [20–23]. In many European countries, internal organs (liver, kidneys, heart, and lungs) are sold and consumed as a valuable food source. Therefore, evaluating toxic metal levels in internal organs is important for safety and health purposes. The risks to health from certain elements in food can be assessed by comparing estimates of dietary exposures with the Provisional Tolerable Weekly Intakes (PTWIs) and Provisional Maximum Tolerable Daily Intakes (PMTDIs) recommended by the Joint Expert Committee on Food Additives (JECFA) of the Food and Agriculture Organization of the United Nations and the World Health Organization International Programme on Chemical Safety [24,25].

In order to ensure the safety of consumers in Serbia, the present study aims to conduct an exposure assessment of toxic elements (As, Cd, Hg and Pb) due to the consumption of pork meat by products in focusing on the presence of the toxic elements in the kidneys of healthy slaughtered swine. Additionally, the toxicological assessment aim for this study is the assessment of the relationship between pathomorphological changes in kidney and the accumulation of toxic elements.

## 2. Results and Discussion

### 2.1. Incidence of Heavy Metals

Regional variations in the occurrence of toxic elements in the kidneys of slaughtered pigs and the number of samples falling into specified concentration ranges are summarized in Table 1. Arsenic was detected in only one sample originating from the Vladimirci area (content 0.001 mg/kg), while Cd detection in the samples varied from 16.6% (Bogatić region) to 40% (Vladimirci region). Cadmium concentrations in the sampled kidneys from the regions under study ranged from 0.05 to 1.23 ng/g. The highest Cd concentration (1.23 ng/g, mean 0.185 ng/g) was found in Vladimirci region in contrast to Bogatić region where the lowest (0.05 ng/g, mean 0.027 ng/g) concentration was found. The occurrence of Hg varied from 23.3% (Vladimirci) to 43.3% (Senta region). The highest Hg level (0.055 ng/g, mean 0.0033 ng/g) was found in Vladimirci region, in comparasion to Senta region where lowest concentration (0.012 ng/g) of Hg was detected. However, the mean concentration (0.0034 ng/g) for Hg was slightly higher in the samples from the Senta region. A statistically significant difference (p < 0.001) was found among the Cd levels in the kidneys from the areas under study.

Results in Table 1 indicate a higher occurrence of Hg in the sampled kidneys (33.3%), when compared with the incidence of Cd (27.7%) in the samples. However, results showed that the individual and mean Cd concentrations were several times higher than the Hg concentrations. As shown in Table 1, all the levels measured for As. Pb and Hg were below the limits recommended by the by European Regulation [26], however the Cd concentration in one kidney exceeded the maximum limit (1 ng/g) established by European Regulation [26].

On the whole, the concentration of these four analyzed toxic elements was very variable in relation to the region where samples were collected. Also, the present study has shown that toxic elements levels in kidney are comparable with those of other countries, especially within Europe [27,28]. The results of this study confirm that Hg, the toxicity of which is well documented, particularly in its organic form, is one of the main environmental contaminants. In contrast, our results showed that the Cd levels were higher than the levels of Hg in the sampled kidneys (Table 1). In only one sample was an excessive concentration of Cd found (>1.0 ng/g). Cadmium, because of its widespread distribution, movement through terrestrial food-chains, and adverse chronic effects in humans, is one of the toxic elements for which maximum acceptable concentrations have most frequently been set. Offal, especially liver and kidney from adult animals can contain relatively high cadmium levels, and concentrations in excess of 50–100 ng/g in animal tissues are considered normal [9].

The low levels of Cd contamination observed in our study can be explained by the utilization of good agricultural practices (GAP), which uses fertilizer low in Cd and restrict disposal of sewage sludge on crop land. In addition the fact that Pb was not detected in the sampled kidneys could be the results of the global reduction in the discharge of this element in environmental media. Stringent environmental legislation has led to a reduction in the discharges of these metals during the last 15 years [29]. The results from the present work indicate that there are significant regional variations in the occurrence of toxic elements in kidneys from slaughtered pigs. Regional differences in soil and cereal cadmium levels are known to be present. Differences in geographical origin of the cereals used by the three different manufacturers might thus explain the concentration differences in corresponding feedstuff formulation, as could differences in the composition of the formulas. A third, less probable explanation, is cadmium contamination during the industrial processes. The main source of human exposure to mercury is the diet. Mercury occurs in food naturally (e.g., in fish which take up mercury from marine sediments), or as a result of pollution (e.g., emissions from industrial processes, fossil fuel combustion). For non-plant feed materials, Scientific Committee on Animal Nutrition (SCAN) [30] identified fishmeal to be the most common source of mercury for farmed animals under normal farming conditions. In addition to mercury in feed materials, livestock and poultry may also be exposed to mercury in drinking water. However, water does not make a significant contribution to the exposure of livestock except in highly polluted areas. Mercury is a naturally occurring element that is also released to the environment by a variety of sources including human activities. Once released into the environment mercury undergoes a series of complex chemical and physical transformations as it cycles between atmosphere, land, and water. Humans, plants, and animals are routinely exposed to environmental mercury and accumulate it potentially resulting in a variety of health effects.

Also, fishmeal normally contains lower cadmium content, but mineral feeds (mono and dicalcium phosphate) are usually structurally contaminated with Cd. Other minerals influence the absorption and tissue disposition of cadmium and *vice versa*. In pigs, a significant increase of cadmium accumulation in tissues could be observed when high dietary supplements of copper were used in commercial pig fattening rations [31]. Moreover, cadmium absorption is increased if dietary calcium is low [32].

However, these findings suggest that industrial activities in the studied areas introduce higher amounts of Cd than Hg into the food chain. Higher Cd concentrations are found in hot spots related to human activities and on agricultural land where high amounts of phosphate fertilizers and manure are utilized [33]. The total concentrations of toxic elements found in the analysed kidneys were usually low and did not exceed the maximum permissible levels established by the Serbian legislation. Since vegetable foods constitute the major source of Cd for humans [1,34] it can be concluded that the levels of total toxic elements including Cd found in the tested kidneys do not represent an imminent toxicological risk to Serbian consumers. However, it is very important to keep the measures necessary to maintain a steady control of the levels of these elements in staple food in the country in view of the potential health hazard that toxic elements represent.

### 2.2. Pathomorphology Examination

**Figure 1.**
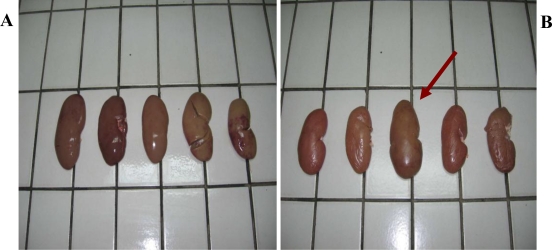
External surface of kidneys from which where Hg and Cd were detected.

**Figure 2.**
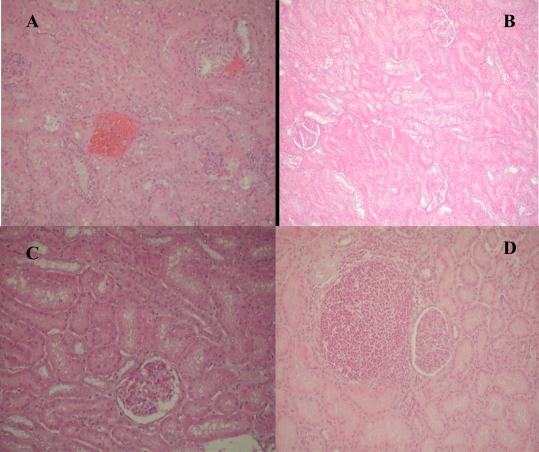
Major renal histopathological changes. Hemorrhages in cortex (A), Fatty change (B), Dystrophy and vacuolar degeneration in the epithelium of proximal tubules` cells (C), and Focal interstitial nephritis (D).

**Figure 3.**
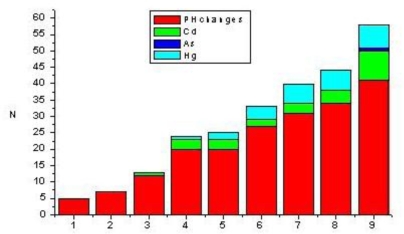
Summary of pathohistological findings (PH) of renal tissues and incidence of toxic elements in kidney from slaughtered pigs (n = 90). Necrosis of proximal tubule cells (1), hypercellularity of vascular loop (2), vascular changes (3), exudat in Bowman’s space (4), focal interstitial nephritis (5), dystrophy of proximal tubule cells (6), swelling of proximal tubule cells (7), renal hemorrhages (8), fatty changes of proximal tubules cells (9). N-number of pathohistological findings of renal tissues and co-incidence of toxic elements in kidney.

The pathomorphological examination results are summarized in Figures 1–3.

#### 2.2.1. Gross pathology

In all 90 pigs were slaughtered during the study period. Kidneys from these pigs submitted to the laboratory were pale, swollen and enlarged with a change in color from the normal mahogany to tan, as follows: 43 (47.7%) had “mottled or pale kidneys”, while 27 (30%) had enlarged kidneys (Figure 1) and 11 (12.2%) were smaller than normal. The only macroscopic lesions observed in few cases were small grey-white foci on the kidney surface. No obvious difference was observed between the right and left kidney. No significant changes were seen in other organs. The external surface of kidney in which Hg (first four) and Cd (the last one) were detected, are shown in Figure 1A, while, the external surface of kidney in which co-incidence of Hg and Cd (0.012 ng/g and 0.05 ng/g, respectively) was detected, is shown in Figure 1B (red arrow).

#### 2.2.2. Pathohistological examination

Histological examination of the kidneys showed two types of changes: degenerative-affecting epithelial cells in some proximal tubules of pigs, and proliferative changes in the interstitium. The major renal histopathological changes were mainly in the epithelium of proximal tubules (Figures 2 and 3). Dystrophy (moderate to marked degenerative changes, Figure 2C), swelling, vacuolization and lipidosi, were the main changes in the tubular epithelial cells. The majority of glomeruli exhibited mild or moderate exudates in Bowman’s capsular spaces as well as hypercellularity of vascular loops. In addition vascular changes expresed as a hyperaemia of blood vessels, moderate to marked haemorrhages of some renal cortical regions occurred occasionally (Figures 2A and 3). In the interstitium of some renal cortical regions, there was limited proliferation of connective tissue (Figure 2D) and focal infiltration of mononuclear inflammatory cells which was sometimes accompanied by small granulomas.

Lipidosis of proximal tubule cells, renal hemorrhages, and swelling of proximal tubule cells were seen in 41 (45.5%), 34 (37.7%) and 31 (34.4%) kidneys of slaughtered pigs, respectively. Twenty seven (30%) of the examined pigs kidneys had a dystrophy of proximal tubules cells, while exudate in Bowman`s space as well as focal interstitial fibrosis were seen in 20 (22.2%) kidneys of slaughtered pigs. Vascular changes expresed as a hyperaemia of blood vessels was seen in 12 (13.3%) kidneys, while hypercellularity of vascular loops, necrosis of proximal tubule cells and renal adenoma occurred in a lesser degree. From a total of 56 analyzed kidneys samples in which the presence of toxic elements was found in 11 samples were determinet co-occurrence of Cd and Hg, while in only one samples is determined co-occurrence of Cd and As (Figure 3).

The kidney is clearly the major target organ of chronic Cd and Hg toxicity [35–38] Accumulated evidence also indicates that kidney is a target organ for As toxicity. Since kidney is the major organ for As elimination and most of the As is rapidly eliminated through the kidney [39,40], renal cells are, thus, exposed to a major portion of the absorbed As dose. After absorption, most of the toxic elements are accumulated in the liver where it induces the production of metallothioneins (MT) a family of low-molecular-weight metal-binding proteins that aid in the intracellular processing of metal ions [41,42]. Metallothioneins exist in most tissues, have a high cysteine content, and hence may be similar to metal chelators in providing heavy metal tolerance and regulating Hg distribution and retention [43–45]. Intracellular metallothionein has also been demonstrated to protect against metal-induced hematotoxicity and immunotoxicity [46]. Metallothionein has been utilized as a sublethal cellular indicator in fish for Hg exposure [47]. When the synthesis of MT becomes insufficient for binding all Cd ions in the liver, Cd not bound to MT produce hepatocyte injury and a Cd–MT complex is released into the bloodstream. The complex in the plasma is then filtered through the glomeruli in the kidney and taken up by the proximal tubular cells [48,49]. Moreover, under certain conditions [e.g., cell–cell interactions], metal-induced synthesis of metallothionein may also have an immunosuppressive effect on T-cell function [50].

The toxic effects of mercury on the kidney are well characterized and include acute tubular necrosis and reduced glomerular filtration rate. In small doses, the S3 segment in the cortico-medullary area is the primary target site. As the dose of mercury is increased, the injury spreads to involve the S1 and S2 segments of the proximal tubules [51]. A more recent study has shown that Cd nephrotoxicity is also associated with alterations in the localization of the tight junction protein claudin-2 in the proximal tubule [52]. Additional studies have shown that *N*-cadherin and its associated proteins may be involved in the nephrotoxic responses to other metals such as Hg [36,53] and bismuth (Bi) [54].

The biochemical mode of action for Hg toxicity has been postulated to involve interaction of Hg with thiol groups of proteins (R–S–Hg+; R–S–Hg–S–R; R<S2>Hg) [55] via either binding or precipitation of SH groups [56]. Hg has a relatively large thiol association constant, thereby enabling this reaction [57]. Despite the thermodynamic stability of metal–SH complexes, they are generally kinetically labile, and hence are rapidly mobile in most biological systems [58]. Although mercuric ions bind only about 1% of the SH groups on the surface of red blood cells, this binding action is substantial enough to inhibit sugar transport and energy production [59].

Consistent with its thiol binding properties, Hg has been shown to preferentially distribute in the lysosomal fraction of rat cells [57] and interact with phospholipid membranes (specifically phosphatidylserine and phosphatidylcholine) [57,60,61]. Furthermore, while Hg can inhibit enzymes with SH groups, and interact with membrane proteins, it can also substitute for zinc in certain zinc-activated enzymes (e.g., carboxypeptidase) [62] or replace metallothionein-bound Zn, Cu, and Cd. The ability of Hg to interact with phospholipids and specific enzyme systems may help explain the cell degeneration, apoptosis and necrosis, and overall toxicity observed in immune system cells. The effects of I-Hg on transduction at cellular membrane channels have been investigated through studies of Hg^2+^. In a study on the effects of inorganic Hg on cell membranes, Liang *et al.* [63] found that Hg^2+^ induced channelopathies in guinea pig sensory cells by impairing K+ channels and changing the permeability of the cell membrane. Leong *et al.* [64], for example, found that Hg2+ ions suppressed neuronal somata sprouting, thus inhibiting neurite growth in snails. It has been reported that inorganic and organic mercury block voltage-activated Ca^2+^ channels in nerve terminals and disrupt ligand-gated ion channels [65,66]. These and other experimental animal studies demonstrate a wide range of biological effects from exposure to different species of Hg [67–70].

Enlarged kidneys are indicative of renal inflammation and proliferative lesions following chronic exposure to Cd [71] or As [72]. The increased kidney injury from Cd and As co-exposure might be due to increased oxidative stress. It has been proposed that both Cd [73–77] and As [76,77] may produce oxidative stress as a cellular mechanism of toxicity. Other recent studies have shown that alterations in the activity of focal adhesion kinase may play a key role in the malignant transformation of cells by arsenic [78,79]. In this regard, Cd and As co-exposure produces significantly more lipid peroxidation in liver and kidney than either inorganic given alone [80].

In regard to aetiology of porcine nephropathy, the production of multiple toxins as is sometimes the environmental situation, presents a problem that has not been sufficiently investigated. Co-incidence of pathohistological findings of renal tissues and toxic elements in kidneys from slaughtered pigs are summarized in Figure 3.

## 3. Experimental Section

### 3.1. Sampling

A total of 90 kidneys from healthy slaughtered swine from three different regions of Serbia were sampled during a six month period. The slaughtered pigs had an average weight of about 105 kg and age of about six months. Sampling in the slaughterhouses consisted of collecting one kidney per pig, from five pigs per farm. All samples were packed in plastic bags, kept in a small freezer (+4 °C) during transport, and immediately transported to the laboratory. Visible fat, connective tissue and major blood vessels were excised and the samples were then homogenized. Sub-samples (10 g approximately) were taken and frozen at −18 °C until analysis.

### 3.2. Digestions

Kidney samples for instrumental analysis were prepared using the method of acid microwave digestion. Samples underwent digestion in a microwave digestion unit (Milestone TC) with temperature control. Heavy metal contents (Pb and Cd) were determined using GF-AAS technique, atomic absorption spectrophotometer-Varian Spectra 220, equipped with Varian-GTA 110 graphite furnace. Mercury (Hg) concentrations in samples were analyzed by hydride generation atomic absorption spectrophotometry at 253.7 nm (HG–AAS, cold vapour technique), using Varian Spectra A220-atomic absorption spectrometer equipped with a Varian VGA-77 hydride generator. Total arsenic (As) content was measured by hydride generation atomic absorption spectroscopy HG-AAS, in flame at 197.3 nm, on the same instrument configuration as mentioned above.

### 3.3. Quality Assurance

Appropriate quality assurance procedures were carried out to ensure reliability of the results. Samples were carefully handled to avoid contamination. Glassware was properly cleaned, and the reagents were of analytical grade. Double distilled deionised water was used throughout the study. Reagents blank determinations were used to correct the instrument readings. Recoveries for all analytes ranged from 95% to 102% and the variation coefficient ranged between 4% and 9%. Quality control procedures included the analysis of a standard reference material (BCR No. 186).

### 3.4. Microscopic Examination

Kidney samples were fixed in 10% neutral buffered formalin and absolute alcohol for 5 to 7 days, processed using standard histological techniques, and stained with hematoxylin and eosin (HE) for light microscopic examination.

### 3.5. Statistical Analysis

Differences in the mean levels of toxic elements contamination across the three groups of positive samples was determined by analysis of variance and then by a Student’s t-test. Additional posttests were applied to evaluate differences among groups with statistically significant variation among means. Differences with *p* values smaller than 0.05 were considered statistically significant.

## 4. Conclusions

It could be concluded that the presence of toxic elements in swine kidneys and consequently, the human exposure to relevant Provisional Maximum Tolerable Daily Intakes (PMTDIs), Provisional Tolerate Weekly Intakes (PTWIs) and Tolerable Daily Intake (TDIs) of the four toxic elements under study are below that those reported by the FAO/WHO, who have set a limit for toxic element intake based on body weight for an average adult (60 kg body weight) [24,25,81]. Thus, the consumption of average amounts of pork meat and meat byproducts which include internal organs (liver, kidneys, heart, and lungs) does not pose a health risk for the consumer. The present study provides data on the prevalence of toxic elements in pigs intended for food which were raised in different regions of Serbia and also provides an assessment on the consumer’s exposure to the toxic elements. Despite the limited number of samples examined in the present study, it is important to notice that even the low levels of toxic elements concentration found in food, may contribute to the toxic elements daily intake. The findings of this study suggests that regular surveys for toxic elements should be done on all food commodities in order to evaluate the possibility of health risks associated with toxic elements exposure, to assure food safety and to protect the consumers from food that might negatively affect their health. Regular surveys and monitoring programmes for toxic elements contents in foodstuffs have been performed for decades by several countries.

In addition to toxicological assessment it is clearly evident from the studies of many authors and the findings of this study that interactions between toxic elements are very important in toxicology. The kidneys are target organ for As, Cd, Hg toxicity. Long-term, even low-level, exposure to this metal leads to kidney damage characterized by tubular dysfunction. Future research should focus on the interactions between these elements in humans exposed to toxic elements occupationally and environmentally.

## Figures and Tables

**Table 1.** Percentage of samples contaminated with each toxic elements and their concentrations in kidneys from slaughtered pigs [ng/g].

**Toxic elements (ng/g)**	**Region**	**Vladimirci**	**Senta**	**Bogatić**	**Total**
	N	30	30	30	90
**Cd**	n	12	7	6	25
	%	40	23.3	16.6	27.7
	*X̄* ± Sd	0.185 ± 0.322^a^	0.022 ± 0.043^b^	0.027 ± 0.064^c^	0.078 ± 0.20
	Max. value	1.23	0.13	0.27	1.23
**As**	n	1	nd	nd	1
	%	3.3	Nd	Nd	3.3
	*X̄* ± Sd	3.3 × 10^−5^ ± 1.8 × 10^−4^	nd	nd	3.3 × 10^−5^ ± 1.8 × 10^−4^
	Max. value	0.001			0.001
**Hg**	n	7	13	10	30
	%	23.3	43.3	33.3	33.3
	*X̄* ± Sd	0.0033 ± 0.01	0.0034 ± 0.0043	0.0025 ± 0.004	0.0031 ± 0,007
	Max. value	0.055	0.012	0.014	0.055

**N**-total number of analyzed samples; **n**- number of contaminated samples; **%**- percentage of samples contaminated with each toxic elements; *X̄* - mean concentration level (ng/g); **Sd**- standard deviation; **nd**- not detected, a:b p < 0.001, a:c p < 0.001.
